# Expressive Suppression and Negative Affect, Pathways of Emotional Dysregulation in Psoriasis Patients

**DOI:** 10.3389/fpsyg.2019.01907

**Published:** 2019-08-21

**Authors:** Cristina Ciuluvica, Mario Fulcheri, Paolo Amerio

**Affiliations:** ^1^Department of Psychological Sciences, Health and Territory (DISPUTer), University G. D’Annunzio Chieti – Pescara, Chieti, Italy; ^2^Clinic of Dermatology, Department of Medicine and Aging Sciences, University G. D’Annunzio Chieti – Pescara, Chieti, Italy

**Keywords:** expressive suppression, negative affect, emotion dysregulation, psoriasis, maladaptive mechanisms

## Abstract

The main goal of this study was to assess the emotion regulation (ER) mechanisms, such as expressive suppression and cognitive reappraisal, in patients with psoriasis, as compared with healthy persons not afflicted by dermatological diseases. Moreover, the study intended to carry on a multidimensional assessment of emotional mechanisms in persons with psoriasis, highlighting the differences between psoriasis patients and healthy participants, in order to identify the specific patterns of emotion dysregulation (ED) in psoriasis. Another goal of the study was to investigate the predictors of ED among different emotional patterns. We presumed that the maladaptive ER mechanisms are higher in psoriasis patients than in the control group and there are specific dysregulation patterns in psoriasis patients as negative emotions tendency. This cross-sectional study was performed on 192 individuals aged between 35 and 75 years (mean age 59). The sample was divided in two groups: the clinical group including 91 patients with a diagnosis of psoriasis vulgaris and the control group including 101 healthy persons. The results of the present study suggest that psoriasis patients more frequently used emotional suppression – a maladaptive ER mechanism – as well as ED patterns – i.e., impulse control difficulties, and nonacceptance of emotional responses. They also displayed trait tendency to a negative emotional response. In fact, in people with psoriasis, the presence of suppression mechanism and negative affect of trait could predict that 35% of patients will show emotional dysregulated patterns, while living with higher levels of ED. The results of our study are important in the clinical practice, helping clinicians to better understand the emotional vulnerability of people that live with psoriatic disease, and to optimize the disease management and patient care in an interdisciplinary approach.

## Introduction

Psoriasis is a chronic, visible, immune-mediated psychocutaneous disease, which can impact considerably the patient’s life quality and their daily functioning (Yadav et al., 2013; França et al., 2017). This skin condition is characterized by circumscribed, erythematous, dry, scaly plaques and has a fluctuating severity over time (Reich, 2012; Brandon et al., 2019). A range of co-morbidities is associated with psoriasis, including cardiovascular disease, diabetes, obesity, metabolic syndrome, and common psychological disorders like depression and anxiety (Picardi et al., 2004; Gisondi et al., 2013; Miller et al., 2013; Lakshmy et al., 2015; Linder et al., 2015).

Several studies have been conducted showing that there is a significant relationship between psychological stress and psoriasis, highlighting that the stress impact may be higher in psoriasis patients compared to other skin conditions (Basavaraj et al., 2011; Rieder and Tausk, 2012; Nelson et al., 2013). There is evidence in the literature showing that psychological stress may have a role in the onset or exacerbation of a variety of skin diseases (Al’Abadie et al., 1994). Devrimci-Ozguven et al. (2002) suggests that stressful incidents occur before the onset of psoriasis flares in approximately 68% of adult patients.

Although the existence of a relationship between stress and the exacerbation of diffuse plaque psoriasis is now well known, the studies on the specific emotional mechanisms are still at the beginning.

There are different conceptualizations of ER/ED. The starting point of view is that ER refers to attempts individuals make to influence which emotions they have, when they have them, and how these emotions are experienced and expressed (Sloan and Kring, 2007). ER involves the pursuit of desired emotional states (i.e., emotional goals) in the service of superordinate motives (Tamir, 2016). A recent definition (Bunford et al., 2015) considers ER as an individual’s ability to modulate: the speed with which and what degree to which the physiological, experiential, and behavioral expression of an emotion escalates, the intensity of physiological, experiential, and behavioral expression of an emotion, and the speed with which and degree to which physiological, experiential, and behavioral expression of an emotion deescalates in a manner congruent with an optimal level of functioning.

In opposition to the definition of ER, Werner and Gross (2010) define ED as “the inability to flexibly respond to and manage emotions.” There are a wide variety of approaches used in the research of the complex phenomena included under the heading of ED. Gratz and Roemer (2004) conceptualized ED as a process that involves four dimensions: awareness and understanding of own emotions, acceptance of own emotions, ability to control impulsive behaviors when leaving negative emotions, and capability to use appropriate ER strategies to achieve desired goals. Based on this conceptualization, the authors defined ED (or the difficulties in ER) as the relative absence of any or all of these abilities, and developed a model of ED in six factors, that we used in the present research. The six ED factors were assessed as the DERS’s six subscales (Difficulties in Emotion Regulation Scale).

In the present work, we used Gross’ conceptual framework of ER who considers the temporal aspects of emotion. In this conceptualization, emotion is a special case of affect, relatively brief and referential (Frijda, 1986; Ekman, 1992). Gross (1998) in describing the ER process model makes the distinction between antecedent- focused (e.g., cognitive reappraisal) and response-focused (e.g., expressive suppression) ER. Antecedent-focused regulation consists of early attempts to modify emotions that occur before the full activation of emotional response. Response-focused regulation consists of late attempts to modify emotion, when the emotional response has already generated. Cognitive reappraisal refers to thinking about a situation in a manner that can alter its emotional response. Expressive suppression occurs when an individual attempts to inhibit the behavior of emotional expression (Gross, 1998).

The aim of this study was to assess the ER mechanisms such as expressive suppression and cognitive reappraisal in patients with psoriasis as compared with healthy persons not afflicted by dermatological diseases. Moreover, the study intended to carry on a multidimensional assessment of emotional mechanisms in persons with psoriasis, highlighting the differences between psoriasis patients and healthy participants, to identify the specific patterns of ED in psoriasis. Following this purpose, different conceptualization of emotion and ER were considered, assessing the type (trait/state) and quality (positive/negative) of emotion (PANAS model known as consensual model), the adaptive/maladaptive ER mechanisms (Gross and John two-factor model), and the ED patterns when living negative emotions (Gratz and Roemer’s six-factor model).

The present research is going to answer whether or not the adaptive and maladaptive ED mechanisms like expressive suppression and cognitive reappraisal in psoriasis patients differ from that of a healthy person. The aim of the study is also to investigate the predictors of ED (among different patterns of the emotional phenomenon). We presume that there are specific dysregulation patterns in psoriasis patients, and the maladaptive ER mechanisms are higher in psoriasis patients than in the control group. We presume also that the difficulties in ER depend not only on the type (trait/state) and quality (positive/negative) of emotion but also on the maladaptive strategies (expressive suppression). In psoriasis patients, the contribution of expressive suppression in ED is higher than in healthy persons. Expressive suppression and cognitive reappraisal in patients with dermatological diseases have never been investigated so far, especially in the context of a multidimensional assessment of the emotional phenomenon.

## Materials and Methods

### Sample

This cross-sectional study was performed on 192 individuals aged between 35 and 75 years (mean age 59). The sample was divided in two groups: the clinical group including 91 patients with a diagnosis of psoriasis vulgaris, and the control group, consisting of 101 healthy volunteers. The study was conducted in the Dermatology and Venereology Clinic, of the “SS Annunziata” University Hospital in Chieti, Italy between October 2017 and January 2019 in accordance with ethical standards on human experimentation and with the Helsinki Declaration of 1975, as revised in 1983.

The patients came in the clinic for an out-patient visit and completed the assessment protocol in the presence of a clinical psychologist with experience in emotion assessment, who was blind regarding the disease severity. The severity of the psoriasis was assessed by a dermatologist. Psoriasis vulgaris was the only type of psoriasis that was considered for the study. All the individuals signed the informed consent form and a questionnaire regarding their demographic data. The diseases characteristics such as illness length, illness severity, and type of medication are presented in Table 1.

**Table 1:** Disease characteristics in psoriasis group: illness length, illness severity, and type of medication.

**Illness length, % (*n*)**	
≤1 year	5.8 (5)
≤5 years	7.4 (8)
≥5 years	86.8 (79)
**Illness severity, % (*n*)**	
PASI < 10	82.6 (75)
PASI ≥ 10	17.4 (16)
DLQI < 10	79.2 (72)
DLQI ≥ 10	20.8 (19)
**Medication, % (*n*)**	
Biological	(12.1) 11
Phototherapy	(19.7) 18
Topical	(44.1) 41
No medication, % (*n*)	(24.1) 21

Inclusion criteria were as follows: age (over 18) and the diagnosis of psoriasis vulgaris. Exclusion criteria were as follows: history of a major neurological disease (dementia – 1) or a major psychiatric condition (anxiety – 2, depression – 1), and the inability to complete the assessment for different reasons (age, culture, education – 3), failure to give informed consent (2).

The control group of healthy participants was matched to the patients by gender, age, and educational qualification, did not present any somatic or psychic disease, and all signed an agreement to participate in this study. The demographical characteristics of the participants are presented in Table 2.

**Table 2:** Participants characteristics.

	*N*	Age in years	Gender	Working status	Civil status	Education (years)
		Mean ± SD	Female *n* (%)	Working *n* (%)	Married *n* (%)	Mean ± SD
Psoriasis	91	49.17 ± 16.54	46 (50.7)	51 (58.2)	61 (67.3)	11.56 ± 2.91
Healthy individuals	101	50.82 ± 10.84	53 (52.3)	62 (62.4)	73 (72.2)	12.48 ± 3.84
Total sample	192	50.08 ± 16.67	99 (58.2)	113 (59.2)	134 (69.4)	12.86 ± 3.92

### Measures

#### Illness Severity Assessment

##### Psoriasis Area Severity Index

Psoriasis Area Severity Index (PASI) was used to establish severity of psoriasis. The human body is divided into four areas separately: head (H) (10% of a person’s skin); arm (A) (20%); trunk (T) (30%); and legs (L) (40%). The four scores are combined into the final PASI score. Also, within each body area, the severity is estimated by three clinical signs: erythema (redness), induration (thickness), and desquamation (scaling). Severity is evaluated on a scale of 0 (none) to 4 (maximum).

The sum of all three severity parameters is then calculated for each section of skin, multiplied by the area score for that area and multiplied by the weight of the respective section. A PASI score < 10 depicts a mild psoriasis while when PASI score is >10 psoriasis is moderate to severe (Augustin et al., 2018). The mean PASI score in our clinical group was 7.62 (SD 8.93).

##### Dermatological Quality Severity Index

Dermatological Quality Severity index (DLQI) designed to measure the health-related quality of life of patients older than 16 suffering from a skin disease. DLQI consists of 10 questions concerning a dermatology patient’s perception of the impact of their skin disease on different aspects of their quality of life (QoL) over the last week, including symptoms and feelings, daily activities, leisure, work or school, personal relationships, and the side effects of treatment. Higher scores indicate greater impairment of QoL. The mean DLQI score in our clinical group was 6.06 (SD 5.62).

#### Psychological Assessment

All patients were asked to complete the following psychological variables: expressive suppression, cognitive reappraisal, ED and its components, positive and negative affect of state, and positive and negative affect of trait. The research protocol included Italian validated versions of several questionnaires: Emotion Regulation Questionnaire (ERQ), Difficulties in Emotion Regulation Scale (DERS, Positive and Negative Affect Schedule of Trait (PANAS of Trait), and Positive and Negative Affect Schedule of State (PANAS of State).

##### Emotion Regulation Questionnaire

Emotion Regulation Questionnaire (ERQ; Gross and John, 2003) was used to measure ER. The ERQ is a 10-item checklist divided into two sub-scales, capturing two commonly used ER strategies, cognitive reappraisal and expressive suppression. Reappraisal refers to thinking differently about a potential stressor event, to manage better the emotional response, whereas suppression refers to diminishing or inhibiting the emotional expression when facing the same emotional event. Participants were required to rate their response on a 7-point Liker-type scale (1 = totally disagree to 7 = totally agree) on their usual ways of emotional regulation. A higher score indicates a higher tendency to adopt one or another strategy. The total score on ERQ indicates the global value of ER. The internal consistency in the present sample was 0.73 for expressive suppression and 0.79 for cognitive reappraisal.

##### Difficulties in Emotion Regulation Scale

Difficulties in Emotion Regulation Scale (DERS; Gratz and Roemer, 2004) is a 36-item multidimensional self-report measure, subdivided into six subscales: (1) *Nonacceptance* of emotional responses; (2) *Goals* (difficulties engaging in goal-directed behavior when experiencing negative emotions); (3) *Impulse* (impulse control difficulties when experiencing negative emotions); (4) *Awareness* (lack of emotional *awareness*); (5) *Strategies* (limited access to ER *strategies* that are perceived as effective); and (6) *Clarity* (lack of emotional clarity), assessing the individual’s characteristic patterns of ED. Items are rated on a 5-point Likert-type scale (from 1 = almost never to 5 = almost always). A high score indicates the presence of a major difficulty in ER. The total score on DERS indicates a global value of ED. A high total score indicates a high level of ED. In the current sample internal consistency ranged between 0.73 for awareness and 0.81 for strategies and acceptance.

##### Positive and Negative Affect Schedule

Positive and Negative Affect Schedule (PANAS; Watson et al., 1988) is a 20-item questionnaire. Ten items measure positive affect (interested, excited, and inspired), and 10 items measure negative affect (e.g., upset, afraid). PANAS was designed to measure affect in various contexts such as at the present moment, the past day, week, or year, or in general (on average). Thus, the scale can be used to measure state affect, dispositional or trait affect, emotional fluctuations throughout specific period, or emotional responses to events. High scores are related to the more frequent use of one kind of emotion.

In the present study, we used two versions of PANAS: PANAS trait, evaluating the emotions that one generally proves (trait tendency to experience positive or negative emotions), and PANAS state, evaluating the emotions that one proves in the evaluation moment (positive or negative emotional reactivity in a specific situation). Results were recorded as: positive affect of trait (PAT), negative affect of trait (NAT), positive affect of state (PAS), and negative affect of state (NAS). The Cronbach’s *α* in the present sample ranged between 0.79 for PAS and 0.86 for NAS.

### Statistical Analysis

Data were entered into a computerized database and analyzed with the SPSS Statistics version 21 (IBM) program. The level of significance was set at *p* < 0.05 and *p* < 0.001. Descriptive analyses were run to determine means, standard deviations, and ranges for all characteristics of both psoriasis and control participants. All variables were checked for the assumption of normal distribution. Bivariate correlations were performed to determine relations between Suppression, Reappraisal, DERS scores, Positive and Negative Affect of Trait, and Positive and Negative Affect of State. In order to verify our hypothesis about differences in mean results between patients with psoriasis and healthy persons in ER mechanisms, the independent samples *t*-test was performed. Standard multiple regression was applied to predict ED from the expressive suppression, negative affect of trait, and negative affect of state, separately for psoriasis patients and healthy individuals. The dependent variable was ED (DERS total score). The independent variables were expressive suppression, negative affect of trait, and negative affect of state.

## Results

### Emotion Regulation Mechanisms in Psoriasis and Healthy Individuals

Our results suggest that psoriasis patients show a higher maladaptive mechanism (expressive suppression) when attempting to regulate their emotions (*Z* = 3.112, *p* < 0.002) compared to healthy controls, where no difference was shown in the adaptive mechanism (cognitive reappraisal) (Table 3).

**Table 3:** Emotion assessment in the two groups (psoriasis patients and controls).

	Psoriasis	Controls		
	(*n* = 91)	(*n* = 101)		
	Mean ± SD	Mean ± SD	*t*	*p*
**Emotion regulation mechanisms**				
Expressive suppression	17.49 ± 5.95	14.70 ± 5.85	3.112	0.002**
Cognitive reappraisal	29.71 ± 7.67	30.37 ± 6.76	−0.624	0.533
Emotion regulation	47.09 ± 10.56	45.16 ± 8.65	1.374	0.171
**Emotion dysregulation**				
Nonacceptance	13.41 ± 6.65	11.39 ± 5.14	2.311	0.022*
Goals	12.41 ± 5.14	12.01 ± 4.02	0.585	0.559
Impulse	12.47 ± 5.15	10.30 ± 4.02	3.193	0.002**
Awareness	6.81 ± 3.20	7.41 ± 3.12	1.255	0.211
Strategies	17.21 ± 5.78	17.16 ± 5.05	0.064	0.949
Clarity	10.44 ± 4.76	9.75 ± 3.18	1.169	0.244
Emotion dysregulation	72.79 ± 23.55	68.01 ± 16.46	1.608	0.110
**Affect quality**				
Positive affect of trait	33.74 ± 7.45	31.49 ± 7.74	1.975	0.052
Negative affect of trait	21.16 ± 9.23	17.65 ± 6.64	2.970	0.003**
Positive affect of state	30.01 ± 8.21	29.11 ± 7.91	0.748	0.456
Negative affect of state	14.62 ± 7.31	15.14 ± 5.38	−0.548	0.585

*Differences between groups*.

* *p < 0.05*;

** *p < 0.005*.

As for the maladaptive regulation, we conclude that patients with psoriasis show major ED patterns as opposed to healthy controls and reported higher scores on two out of six dimensions of DERS: nonacceptance (*Z* = 2.311, *p* = 0.022) and impulse (*Z* = 3.193, *p* = 0.002) (Table 3).

The PANAS score was consistently higher for psoriasis patients, compared to normal subjects on only one-dimension NAT (negative affect of trait). There was no difference between the two groups regarding negative or positive affect of state and positive affect of trait (Table 3).

To better delineate the relation between disease severity and the mechanism of ED, we performed a statistical analysis dichotomizing patients into two groups (PASI ≥ 10 and PASI < 10). We found no differences between the two groups.

### Correlations Between Variables

When we tried to correlate ED and positive and negative affect in psoriasis patients, we found that negative affect of trait was strongly related with ED (0.472, *p* < 0.001, while negative affect of state was only moderately correlated (0.27, *p* < 0.05). DERS also correlated with suppression mechanism of emotion control (0.28, *p* < 0.001). Moderate correlation was found between DERS and disease severity (0.37, *p* < 0.05). Quality of life negatively correlated in psoriasis patients with positive affect of trait (−0.47, *p* < 0.001) (Table 4).

**Table 4:** Correlations between variables in psoriasis group: S (expressive suppression), R (cognitive reappraisal), ER (emotion regulation measured as total score of ERQ), PAT (positive affect of trait), NAT (negative affect of trait), PAS (positive affect of state), NAS (negative affect of state), ED (emotion regulation measured as total score of DERS), AGE, TIME (illness length), PASI (disease severity), and DLQI (quality of life). The relevant values are presented in bold format.

	1	2	3	4	5	6	7	8	9	10	11
1.ED	–										
2.AGE	−0.015	–									
3.S	**0.312** **	−0.026	–								
4.R	0.011	0.009	−0.169	–							
5.ER	0.185	0.004	0.685**	0.828**	–						
6.PAT	−0.193	−0.038	−0.072	**0.338** **	0.205	–					
7.NAT	**0.497** **	−0.154	−0.017	−0.137	−0.111	**0.217** *	–				
8.PAS	−0.074	0.066	0.143	0.080	−0.039	0.688**	0.296**	–			
9.NAS	**0.300** **	−0.153	−0.017	−0.092	−0.078	0.010	0.669**	0.056	–		
10.DLQI	0.167	−0.233	−0.104	−0.209	−0.204	**−0.464** **	−0.140	−0.220	0.076	–	
11.PASI	0.347	−0.168	0.086	−0.045	0.011	−0.069	0.056	0.117	0.138	0.504**	–
12.TIME	0.145	**0.540** *	−0.224	0.044	−0.093	0.132	0.333	0.062	0.204	−0.226	−0.12

* *p < 0.05*;

** *p < 0.01 (two-tailed)*.

In healthy controls, there was a positive moderate correlation between suppression and DERS (0.235, *p* < 0.05) and DERS resulted negatively correlated with positive affect of trait (−0.29, *p* < 0.001), and positively related with negative affect of trait (0.37, *p* < 0.001) (Table 5).

**Table 5:** Correlations between variables in control group: S (expressive suppression), R (cognitive reappraisal), ER (emotion regulation measured as total score of ERQ), PAT (positive affect of trait), NAT (negative affect of trait), PAS (positive affect of state), NAS (negative affect of state), ED (emotion dysregulation measured as total score of DERS), and AGE. The relevant values are presented in bold format.

	1	2	3	4	5	6	7	8
1. ED	–							
2. AGE	0.129	–						
3. S	**0.235** *	0.082	–					
4. R	−0.129	0.048	−0.065	–				
5. ER	0.058	0.093	0.626**	0.738**	–			
6. PAT	**−0.291** **	−0.161	−0.123	**0.237** *	0.102	–		
7. NAT	**0.377** **	−0.182	0.019	−0.156	−0.110	0.096	–	
8. PAS	**−**0.083	0.035	−0.071	**0.388** **	**0**.**255** **	0.756**	0.043	–
9. NAS	0.177	0.017	0.035	−0.068	−0.029	0.031	0.629**	0.172

* *p < 0.05*;

** *p < 0.01 (two-tailed)*.

There were no significant correlations between DERS and all other variables (e.g., age and gender) in both study groups.

### Predictors of the Emotion Dysregulation in Psoriasis and Healthy Group

In order to investigate the ED’s predictors (among ER mechanisms, quality, and type of emotions), a multiple linear regression was run, considering ED as dependent variable, and expressive suppression, negative affect of trait, and negative affect of state as independent variables. Results of the multiple regression in psoriasis group suggest that these variables statistically significantly predicted ED, *F*(3, 89) = 13,860, *p* < 0.0005, *R*^2^ = 0.35, which indicates that 35% of ED variance is explained by our model. Inspection of the regression coefficients indicates that only two variables of three are significant predictors of level of ED in psoriasis: expressive suppression (*β* = 0.304, *p* < 0.001) and negative affect of trait (*β* = 0.557, *p* < 0.001) (Table 6).

**Table 6:** Standard regression analysis summary for psoriasis patients’ variables predicting emotion dysregulation.

Variables	*B*	SEB	*β*
Expressive suppression	1.169	0.352	0.304^**^
Negative affect of trait	1.391	0.309	0.557^**^
Negative affect of state	−0.216	0.389	−0.069

*R^2^ = 0.35 (*N* = 89, *p* < 0.001)*.

** *p < 0.001*.

Psoriasis patients with the tendency to negatively respond to emotional stressors and who control their emotions by not expressing it, especially by suppressing the behavioral expression are more likely to present a higher level of ED, than patients who do not use these mechanisms.

In healthy controls, these variables statistically significantly predicted ED, *F*(3, 97) = 8,146, *p* < 0.0005, *R*^2^ = 0.19, which indicates that only 19% of ED variance is explained by the model. Inspection of the regression coefficients indicates that the same two variables of three are significant predictors of level of ED in healthy persons: expressive suppression (*β* = 0.230, *p* < 0.05), and negative affect of trait (*β* = 0.441, *p* < 0.001).

The results suggest that negative affect of trait is the strongest predictor of ED both in psoriasis and control group, followed by expressive suppression. There are two important differences between the two groups regarding the prediction model of ED: the model explains the higher ED variance in psoriasis patients (35%) when compared to healthy persons (19%), and the contribution of expressive suppression is moderately significant in healthy persons (*p* < 0.05), and quite significant in psoriasis patients (*p* < 0.001) (Table 7).

**Table 7:** Standard regression analysis summary for healthy individuals’ variables predicting emotion dysregulation.

Variables	*B*	SEB	*β*
Expressive suppression	0.648	0.256	0.230^*^
Negative affect of trait	1.093	0.290	0.441^**^
Negative affect of state	−0.331	0.358	−0.108

*R^2^ = 0.19 (*N* = 101, *p* < 0.001)*.

* *p < 0.05*;

** *p < 0.001*.

## Discussion

ER, the experiencing, processing, and modulating of emotional responses, is necessary to manage the emotional stressors common in patients with chronic illness. The present research intended to realize a multidimensional assessment (adaptive and maladaptive emotional mechanisms, ED and its components, quality and type of affect) in subjects affected by psoriasis, highlighting the differences with healthy controls. The study tried to investigate different emotional mechanisms that could better reflect both psychosomatic and somatopsychic perspective on the emotional impact in chronical illness.

The studies on the ER in chronical illness are still at the beginning and scarce in number. Among these studies, only a few are assessing the role of expressive suppression and cognitive reappraisal mechanisms (Wierenga et al., 2017). As far as we know, the present study is the first to investigate, in a multidimensional assessment, the expressive suppression mechanism in psoriasis.

Our study revealed that patients with psoriasis significantly differ from healthy individuals in expressive suppression, negative affect of trait, nonacceptance of own emotions, and in difficulties in controlling their impulses. There is no difference between the two groups in the use of adaptive ER mechanisms. People with psoriasis use more frequently a maladaptive regulation mechanism, trying to modify their emotion, aiming to suppress the behavior of emotional expression.

Regarding the quality of emotions, our study suggests that the difference between psoriasis patients and healthy controls is more pronounced as for negative emotions of trait meaning that psoriatic patients experience more frequently negative emotions. The existing literature supports the presumption that higher negative affect in psoriasis patients could be relate to higher expressive suppression. John and Gross (2004) suggests that “re-appraisers” experience and express greater positive emotion and lesser negative emotion, whereas “suppressors” experience and express lesser positive emotion, yet experiencing greater negative emotions.

Cognitive reappraisal and expressive suppression are two emotional regulation mechanisms that directly influence the physiologic body response (e.g., reappraisal decreases the cardiac pressure. Suppression is a maladaptive emotional regulation mechanism which has been shown to produce sympathetic activation, and may substitute an appropriate control by increasing the emotional arousal. Literature suggests that the biological mechanism of suppression and stress have common components, such as cortisol release (Gross and Levenson, 1993, 1997). At the same time, acute psychologic stress is associated with increased glucocorticoid levels, adversely affecting skin barrier function recovery, induced by tape stripping (Lams et al., 2009). This induced barrier alteration function could explain the stress impact in onset, development, exacerbation of psoriatic plaques in persons affected by psoriasis.

In line with previous researches, our study suggests the existence of higher ED patterns in patients with psoriasis (Ciuluvica et al., 2014; Innamorati et al., 2016; Almeida et al., 2017). Patients reported more problems than healthy controls with the acceptance of emotions, and in controlling their impulses when experiencing negative emotions. Patients with more severe disease cases are more likely to show a dysregulated emotional mechanism.

The results verified the hypothesized model of prediction of ED. In psoriatic patients, the presence of a suppression mechanism and negative affect of trait (the trait tendency of emotional response) could predict that 35% of patients will show emotion dysregulated patterns, experiencing higher levels of emotional dysregulation. There are different results in the healthy controls model because, as our results suggest, the ED is predicted only in 20% by the model. Suppression is only a highly significant predictor of ED in psoriasis patients. In healthy individuals, the significance of suppression as predictor of ED is moderate.

The results suggest that there are emotional patterns in psoriasis patients (expressive suppression, tendency to experience more negative emotions, nonacceptance of own emotions) that may lead to an increase of negative affectivity, forcing patients to supplement their efforts in managing the negative emotional arousal that impacts the wellbeing and life quality.

Indeed, the results of our study also suggest that quality of life in psoriatic patients is significantly correlated with negative affectivity. However, at this point of the study, we are not able to conclude the decisive role of ER in the etiology of psoriasis, but our results suggest the existence of an emotional vulnerability in people affected by psoriasis. Negative emotions that last too long, excessive suppression of emotions, especially when paired with lack of behavioral expression can, through the vegetative nervous system, deregulate the hormonal and immunological systems, cause somatic dysfunction, and finally lead to disease or aggravate the disease course.

The results of the present study suggest that psoriasis patients used a maladaptive ER mechanism (emotional suppression) more frequently, experienced more negative emotions of trait (trait tendency to a negative emotional response), and use emotional dysregulation patterns more frequently: impulse control difficulties, and nonacceptance of emotional responses. The relationship between these specific emotional patterns, the diseases particularities, and their implication in the disease control should be analyzed in more detail in future research because these findings could help physicians to better manage the disease with regard to QoL.

## Data Availability

The datasets generated for this study are available on request to the corresponding author.

## Author Contributions

This manuscript updates and extends a preliminary research report presented at the 6th Annual Scientific Conference of the European Association of Psychosomatic Medicine (EAPM) 2018. The final version of this manuscript was written by CC, PA, and MF. CC and PA contributed to the collection and analysis of psychological and medical data and wrote a preliminary version of this manuscript.

### Conflict of Interest Statement

The authors declare that the research was conducted in the absence of any commercial or financial relationships that could be construed as a potential conflict of interest.

## References

[ref1] Al’Abadie M. S. Kent G. G. Gawkrodger D. J. (1994). The relationship between stress and the onset and exacerbation of psoriasis and other skin conditions. Br. J. Dermatol. 130, 199–203. 10.1111/j.1365-2133.1994.tb02900.x, PMID: 8123572

[ref2] Almeida V. Taveira S. Teixeira M. Almeida I. Rocha J. Teixeira A. (2017). Emotion regulation in patients with psoriasis: correlates of disability, clinical dimensions, and psychopathology symptoms. Int. J. Behav. Med. 24, 563–570. 10.1007/s12529-016-9617-0, PMID: 27924553

[ref3] Augustin M. Langenbruch A. Gutknecht M. Reich K. Körber A. Maaßen D. (2018). Definition of psoriasis severity in routine clinical care: current guidelines fail to capture the complexity of long-term psoriasis management. Br. J. Dermatol. 179, 1385–1391. 10.1111/bjd.17128 [Epub 2018/10/17]. 30334253

[ref5] Basavaraj K. H. Navya M. A. Rashmi R. (2011). Stress and quality of life in psoriasis: an update. Int. J. Dermatol. 50, 783–792. 10.1111/j.1365-4632.2010.04844.x, PMID: 21699511

[ref6] Brandon A. Muffi A. Gary Sibbald R. (2019). Diagnosis and management of cutaneous psoriasis: a review. Adv. Skin Wound Care 32, 58–69. 10.1097/01.ASW.0000550592.08674.43, PMID: 30653184

[ref7] Bunford N. Steven W. E. Wymbs F. (2015). ADHD and emotion dysregulation among children and adolescents. Clin. Child Fam. Psychol. 18, 185–217. 10.1007/s10567-015-0187-5, PMID: 26243645

[ref8] Ciuluvica C. Amerio P. Fulcheri M. (2014). Emotion regulation strategies and quality of life in dermatologic patients. Soc. Behav. Sci. 127, 661–665. 10.1016/j.sbspro.2014.03.331

[ref210] Devrimci-Ozguven H. Kundakci T. N. Kumbasar H. Boyvat A. (2002). The depression, anxiety, life satisfaction and affective expression levels in psoriasis patients. J. Eur. Acad. Dermatol. Venereol. 14, 267–271. 10.1046/j.1468-3083.2000.00085.x, PMID: 11204514

[ref9] Ekman P. (1992). An argument for basic emotions. Cognit. Emot. 6, 169–200.

[ref10] França K. Castillo D. E. Roccia M. G. Lotti T. Wollina U. Fioranelli M. (2017). Psychoneurocutaneous medicine: past, present and future. Wien. Med. Wochenschr. 167(Suppl. 1), 31–36. 10.1007/s10354-017-0573-3 28616665

[ref11] Frijda N. H. (1986). The emotions. Cambridge, UK: Cambridge University Press.

[ref13] Gisondi P. Cazzaniga S. Chimenti S. Giannetti A. Maccarone M. Ricardo M. (2013). Metabolic abnormalities associated with initiation of systemic treatment for psoriasis: evidence from the Italian Psocare Registry. J. Eur. Acad. Dermatol. Venereol. 27, e30–e41. 10.1111/j.1468-3083.2012.04450.x 22313340

[ref14] Gratz L. K. Roemer L. (2004). Multidimensional assessment of emotion regulation and dysregulation: development, factor structure, and initial validation of the difficulties in emotion regulation scale. J. Psychopathol. Behav. Assess. 26, 41–54. 10.1023/B:JOBA.0000007455.08539.94

[ref15] Gross J. J. (1998). Antecedent, and response – focused emotion regulation: divergent consequences for experience, expression, and physiology. J. Pers. Soc. Psychol. 74, 224–237. 10.1037/0022-3514.74.1.224, PMID: 9457784

[ref16] Gross J. J. John O. P. (2003). Individual differences in two emotion regulation processes: implications for affect, relationships and well-being. J. Pers. Soc. Psychol. 85, 348–362. 10.1037/0022-3514.85.2.348, PMID: 12916575

[ref17] Gross J. J. Levenson R. W. (1993). Emotion suppression: physiology, self-report, and expressive behavior. J. Pers. Soc. Psychol. 64, 970–986. 10.1037/0022-3514.64.6.970, PMID: 8326473

[ref18] Gross J. J. Levenson R. W. (1997). Hiding feelings: the acute effects of inhibiting negative and positive emotion. J. Abnorm. Psychol. 106, 95–103. 10.1037/0021-843X.106.1.95, PMID: 9103721

[ref19] Innamorati M. Quinto M. R. Imperatori C. Lora V. Graceffa D. Fabbricatore M.. (2016). Compr. Psychiatry 70, 200–208. 10.1016/j.comppsych.2016.08.001, PMID: 27565774

[ref220] John O. P. Gross J. J. (2004). Healthy and unhealthy emotion regulation: personality processes, individual differences, and life span development. J. Pers. 72, 1301–1333. 10.1111/j.1467-6494.2004.00298.x, PMID: 15509284

[ref20] Lakshmy S. Balasundaram S. Sarkar S. Audhya M. Subramaniam E. (2015). A cross-sectional study of prevalence and implications of depression and anxiety in psoriasis. Psychol. Med. 37, 434–440. 10.4103/0253-7176.168587 PMC4676211 26702177

[ref21] Lams S. Dickerson S. S. Zocola P. M. Zaldivar F. (2009). Emotion regulation and cortisol reactivity to a social-evaluative speech task. Psychoneuroendocrinology 34, 1355–1362. 10.1016/j.psyneuen.2009.04.006, PMID: 19464808

[ref23] Linder D. Altomare G. Amato S. Amerio P. Balato N. Campanati A. (2015). PSOCUBE, a multidimensional assessment of psoriasis patients as a both clinically/practically sustainable and evidence-based algorithm. J. Eur. Acad. Dermatol. Venereol. 29, 1310–1317. 10.1111/jdv.12809 25370415

[ref24] Miller I. M. Ellervik C. Yazdanyar S. Jemec G. B. (2013). Meta-analysis of psoriasis, cardiovascular disease, and associated risk factors. J. Am. Acad. Dermatol. 69, 1014–1024. 10.1016/j.jaad.2013.06.053 24238156

[ref25] Nelson P. A. Chew-Graham C. A. Griffiths C. E. Cordingley L. (2013). Recognition of need in health care consultations: a qualitative study of people with psoriasis. Br. J. Dermatol. 168, 354–361. 10.1111/j.1365-2133.2012.11217.x, PMID: 22880951

[ref26] Picardi A. Amerio P. Baliva G. Barbieri C. Teofoli P. Bolli S. (2004). Recognition of depressive and anxiety disorders in dermatological outpatients. Acta Derm. Venereol. 84, 213–217. 10.1080/00015550410025264 15202838

[ref27] Reich K. (2012). The concept of psoriasis as a systemic inflammation: implications for disease management. J. Eur. Acad. Dermatol. Venereol. 26, 3–11. 10.1111/j.1468-3083.2011.04410.x 22356630

[ref28] Rieder E. Tausk F. (2012). Psoriasis, a model of dermatologic psychosomatic disease: psychiatric implications and treatments. Int. J. Dermatol. 51, 12–26. 10.1111/j.1365-4632.2011.05071.x, PMID: 22182372

[ref29] Sloan D. M. Kring A. M. (2007). Measuring changes in emotion during psychotherapy: conceptual and methodological issues. Clin. Psychol. Sci. Pract. 14, 302–322. 10.1111/j.1468-2850.2007.00092.x

[ref30] Tamir M. (2016). Why do people regulate their emotions? A taxonomy of motives in emotion regulation. Personal. Soc. Psychol. Rev. 20, 199–222. 10.1177/1088868315586325 26015392

[ref32] Watson D. Clark L. A. Tellegen A. (1988). Development and validation of brief measures of positive and negative affect: the PANAS scales. J. Pers. Soc. Psychol. 54, 1063–1070. 10.1037/0022-3514.54.6.1063, PMID: 3397865

[ref33] Werner K. Gross J. J. (2010). “Emotion regulation and psychopathology: a conceptual framework” in Emotion regulation and psychopathology: A transdiagnostic approach to etiology and treatment. eds. Kring A. M. Sloan D. M. (New York: Guilford Press), 13–37.

[ref34] Wierenga K. L. Lehto R. H. Given B. (2017). Emotion regulation in chronic disease populations: an integrative review. Res. Theory Nurs. Pract. 31, 247–271. 10.1891/1541-6577.31.3.247, PMID: 28793948 PMC5992894

[ref35] Yadav S. Narang T. Kumaran S. M. (2013). Psychodermatology: a comprehensive review. Indian J. Dermatol. Venereol. Leprol. 79, 176–192. 10.4103/0378-6323.107632, PMID: 23442456

